# Enhancing microbial metabolite and enzyme production: current strategies and challenges

**DOI:** 10.3389/fmicb.2014.00718

**Published:** 2014-12-18

**Authors:** Koichi Tamano

**Affiliations:** Bioproduction Research Institute, National Institute of Advanced Industrial Science and Technology (AIST)Sapporo, Japan

**Keywords:** productivity enhancement, metabolite, enzyme, metabolic engineering, genome sequencing, bioinformatics, microorganisms

The metabolites and enzymes synthesized by microorganisms have been widely used as food (Mitsuhashi, 2014; Wendisch, 2014), pharmaceuticals (Elander, 2003; Endo, 2010), biofuels (Geddes et al., 2011), pesticides (Waldron et al., 2001; Yoon et al., 2004), and detergents (Shaligram and Singhal, 2010), as well as in the manufacturing process of these industrial products (Kirk et al., 2002; Merino and Cherry, 2007). They play important roles in our daily lives. The production methods used for useful metabolites and enzymes have improved since the time their importance was first established.

If the genes involved in the synthesis of a metabolite or enzyme of interest are unknown, the production yield is enhanced by introducing random mutations into the chromosomes of the synthesizing microbe by ultraviolet (UV) irradiation or treatment with mutagens (Adrio and Demain, 2006). In addition, culture conditions have been adapted to further enhance production (Demain, 2000; Mukherjee et al., 2006). On the other hand, if the genes involved are known, their expression is also enhanced by metabolic engineering strategies such as gene disruption and overexpression using genetic modification techniques (Stephanopoulos et al., 1998; Adrio and Demain, 2010). When genetic modification of the producing microorganism is not possible because of difficulties in transformation, heterologous expression of the product of interest in other microbial species in which genetic modification can be more easily achieved has also been utilized for mass production (Stephanopoulos et al., 1998; Keasling, 2012).

Primary metabolites essential for the normal growth of organisms are conserved between closely related microbial species, and their metabolic pathways including genetic components are almost fully elucidated. Therefore, metabolic engineering has been the chosen strategy used for increasing the microbial production of primary metabolites (Stafford and Stephanopoulos, 2001; Kern et al., 2007). About microbial enzymes, the coding genes are highly likely to be identified if both N-terminal amino acid sequences and molecular weights are not only identified by using highly purified samples but the genomic data of the producer microorganisms are also available. Searching a gene from the genomic data, on the basis of the N-terminal amino acid sequence and molecular weight, will help us identify an enzyme-coding gene. Once the gene has been identified, inducing overexpression of this gene in the original producer or another microbial host is one of the strategies adopted to increase the production of the enzyme (Demain and Vaishnav, 2009).

Four strategies are considered to be effective in enhancing the production of primary metabolites. The first strategy is enhancing the expression of genes involved in metabolite synthesis. This strategy should be the most commonly used and reliable approach, but it does not always contribute to elevated production. In fact, we enhanced the expression of four enzyme genes, individually, that were involved in palmitic acid [C16-fatty acid] synthesis, aiming to increase the production of free fatty acids (primary metabolites) in *Aspergillus oryzae* (Figure 1A-①). Overexpression of the fatty acid synthase (FAS) genes yielded a maximal increase in fatty acid production that was 2.8-fold more than that in the wild-type strain, whereas overexpression of the acetyl-CoA carboxylase (ACC) gene did not increase fatty acid production (Tamano et al., 2013). Overexpression of the two genes encoding ATP-citrate lyase (ACL) and palmitoyl-ACP thioesterase (TES) showed a moderate increase in fatty acid production (Tamano et al., 2013). Thus, each metabolic pathway is believed to consist of many sequential enzyme reactions, one of which has the lowest reaction rate and functions to regulate the rate of the whole pathway like a bottleneck. If an overexpressed gene encodes an enzyme that does not correspond to the bottleneck, there would be no resultant effect on production. In many cases, the bottleneck reactions are unknown; therefore, it is necessary to overexpress each gene involved in the synthesis of metabolites and determine which gene encodes for enzymes functioning at the bottleneck point of the pathway. Alternatively, one may simultaneously overexpress all genes involved in the synthesis in one cell; however, this is a difficult task to accomplish because it is time-consuming and labor-intensive to construct the mutant cell through DNA recombination technology. The second strategy by which primary metabolite production can be increased is the knockout of a reaction that degrades or converts the target metabolites. In *Escherichia coli*, a large amount of fatty acids was successfully produced by a combination of overexpression of genes involved in synthesis and knockout of genes involved in degradation (Steen et al., 2010) (Figure 1A-①,②). The third strategy is to increase the production of the coenzymes required for the synthesis of the target primary metabolites. If sufficient amounts of coenzymes such as ATP, NADH, and NADPH are not synthesized by the producing microorganism, the production yield of the target metabolite as an end product would not increase significantly. Therefore, genetic modification should be used to increase the production of the coenzymes. In fact, the production yield of fatty acids was elevated by increasing the intracellular NADPH molecules available for use in fatty acid synthesis by overexpression of the malic enzyme (ME) gene in *Mucor circinelloides* (Zhang et al., 2007) (Figure 1A-③). The fourth strategy of increasing primary metabolite production is discharging the final metabolites out of the cells. Intracellular accumulation of the final metabolite would stress the producing microorganism, and could possibly have a growth-inhibiting effect on them. In that case, if final metabolites could be discharged from the microorganism as a result of genetic modification and improved culture conditions, target metabolites would continue to be generated because the cell is free from the burden of metabolites accumulating within it. For example, ricinoleic acid, a hydroxylated fatty acid that can be used as an alternative raw material for various petrochemical industrial products, was secreted as a result of overexpression of phospholipase A gene in *Schizosaccharomyces pombe* (Yazawa et al., 2013). Ricinoleic acid was produced continuously during the culture period and it accumulated in the culture supernatant at a concentration that was ~ 10-fold higher than that observed in the absence of overexpression of phospholipase A gene (Yazawa et al., 2013, 2014) (Figure 1A-④).

**Figure 1:**
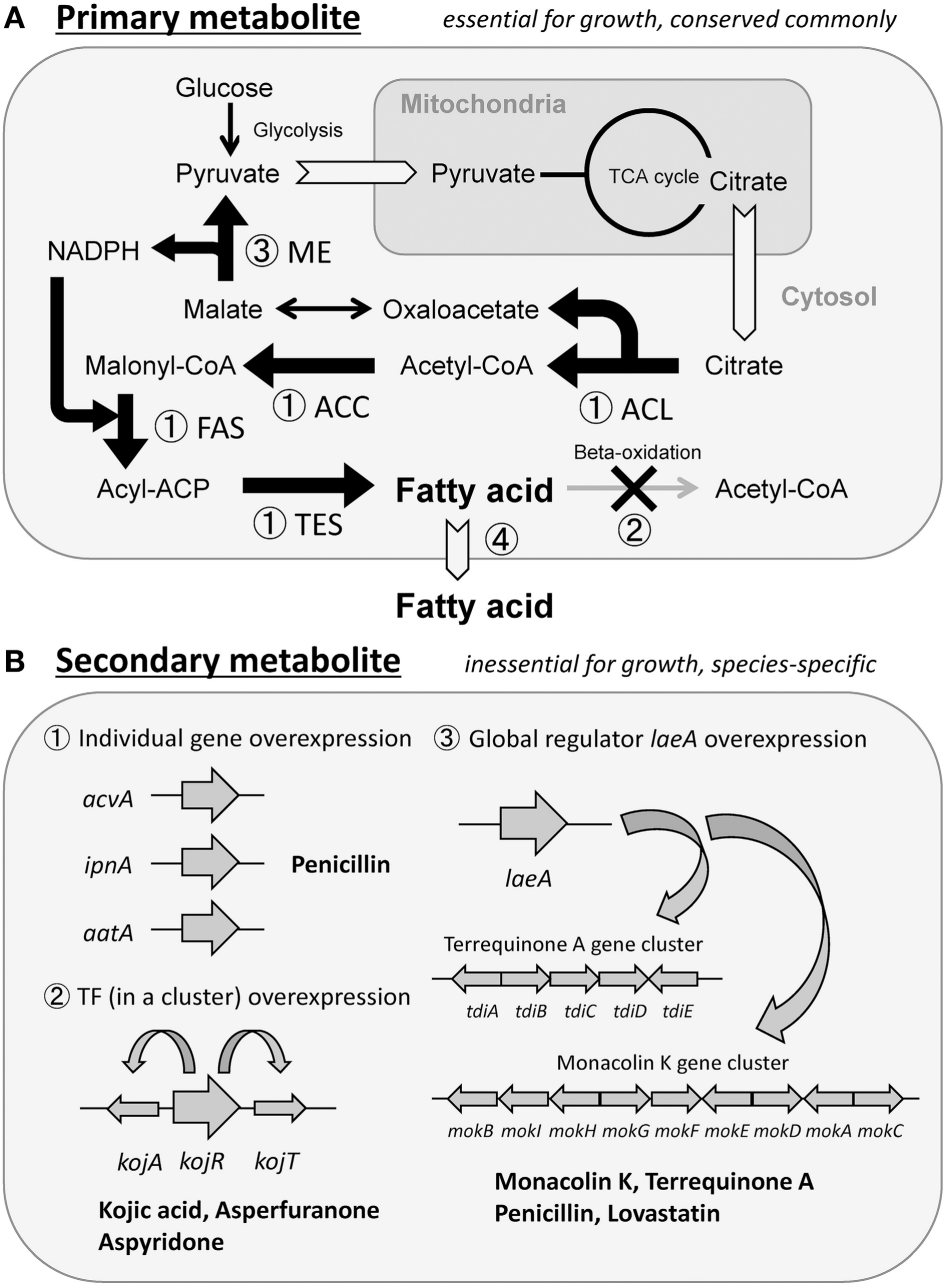
**Strategy to increase production of primary (A) and secondary (B) metabolites**.

An increase in enzyme production is often achieved by overexpressing the genes encoding the enzymes with the help of promoters of constitutively expressed genes or inducible genes in the presence of specific inducer molecules (e.g., IPTG). Enzyme-producing microorganisms are considered to be best for the overexpression strategy because they are equipped with production systems composed of chaperones, foldases (e.g., protein disulfide isomerase), and transporters in addition to the enzyme-coding gene. If overexpression is difficult in the original strain, heterologous overexpression is used as an alternative. Several Gram-negative (e.g., *E. coli*) and Gram-positive bacteria (e.g., *Bacillus subtilis*, *Lactobacillus lactis*), yeasts (e.g., *Saccharomyces cerevisiae*, *Pichia pastoris*, *Hansenula polymorpha*, *Yarrowia lipolytica*), and filamentous fungi (e.g., *A. oryzae*, *Aspergillus niger*, *Trichoderma reesei*) are commonly used for heterologous overexpression (Liu et al., 2013). However, if heterologous overexpression adversely affects the microorganisms, the enzymes would not be produced or their inclusion bodies would form (only in case of *E. coli*).

In case of secondary metabolites, which do not play a role in the normal growth cycle of microbes and are produced by secondary metabolic pathways functioning independently from the primary metabolic pathway, the genetic components are mostly unidentified to date. Even if their molecular structures can be identified by NMR and mass spectrometry using purified samples, it is usually difficult to identify the genes involved in their synthesis from these molecular structures. This could be attributed to the fact that there is no general rule that associates the structure of a secondary metabolite with the DNA sequence involved in the synthesis. Exceptionally, in both polyketide synthases (PKS) and non-ribosomal peptide synthetases (NRPS), the relationship between the amino acid sequence of the functional domain and the structure of the produced secondary metabolite has been elucidated (Finking and Marahiel, 2004; Jenke-Kodama et al., 2005; Donadio et al., 2007). However, many pharmaceutical agents and pesticides of microbial origin are secondary metabolites (Misiek and Hoffmeister, 2007). Therefore, the genes involved in their syntheses are largely unknown, and to enhance their production, conventional strategies such as random mutagenesis are still used predominantly despite the inefficiency in screening mutants showing enhanced production from randomly mutagenized clones.

Between 2005 and 2007, second-generation genome sequencers were launched by three manufacturers, as follows: the SOLiD system by Life Technologies; the Solexa system by Illumina (the system is replaced with the subsequent ones named HiSeq and MiSeq); the Genome Sequencer FLX system by Roche. These systems enabled more rapid and less labor-intensive sequencing of microbial genomes, compared to the conventional system. Thus, genomic DNA of many microbial species and strains were sequenced and registered in public databases. However, simply knowing DNA sequences does not allow identification of the genes involved in secondary metabolite synthesis because of the above-discussed reasons. Thus, it is necessary to somehow predict the genes involved in synthesis from the data that already exist. Three strategies can be potentially used to predict the synthesizing genes. The first is genome-wide comparison of the genetic components between the producing and non-producing strains of the same microbial species. The gene cluster involved in cyclopiazonic acid biosynthesis by the fungus *A. oryzae* was identified using this strategy (Tokuoka et al., 2008). A tool for the prediction of secondary metabolite gene cluster was developed using the comparative genomics approach (Takeda et al., 2014). The second is referring to the genetic functional characteristics of PKS and NRPS predicted by web applications that search for sequence homology (e.g., BLAST). The PKS/NRPS gene involved in the synthesis of the aspoquinolones A–D by the fungus *Aspergillus nidulans* was identified using this strategy (Scherlach and Hertweck, 2006). Using this strategy, four tools such as “SMURF,” “anti-SMASH,” and “CLUSEAN” were developed for the prediction of gene clusters involved in secondary metabolite biosynthesis in filamentous fungi (Weber et al., 2009; Khaldi et al., 2010; Medema et al., 2011; Andersen et al., 2013; Blin et al., 2013). The third strategy is genome-wide profiling using “Omics” information such as that obtained from the transcriptome, proteome, and metabolome. Comparing “Omics” data of the same strain while it is in the producing condition vs. the non-producing condition is considered to be highly effective in identifying the target gene. The gene cluster responsible for kojic acid biosynthesis in the genome of *A. oryzae* was discovered using this method (Terabayashi et al., 2010). Based on the strategy, a program named “MIDDAS-M” was constructed for the prediction of gene clusters involved in secondary metabolite production (Umemura et al., 2013). Using the strategy, a secondary metabolite gene cluster that had neither PKS nor NRPS genes was found in the *Aspergillus flavus* genome, which led to the identification of the gene cluster involved in the biosynthesis of the secondary metabolite ustiloxin (Umemura et al., 2014). Reverse genetics experiments such as gene knockout and successive complementation are necessary to confirm that the predicted genes function in synthesizing the product of interest. If the genes involved in synthesizing secondary metabolites can be identified by these trials, genetic modification could be used to increase metabolite production.

Since secondary metabolites are final products and do not seem to be prone to conversion or degradation by the producing microorganisms, they should be stable after synthesis. Furthermore, since they are usually secreted out of cells, the producing microorganism would be free from the stress of their accumulation. For these reasons, it seemed highly feasible to overproduce secondary metabolites by genetic modification. For example, it is known that in *A. oryzae*, the secondary metabolite penicillin is synthesized in small amounts and secreted. When three enzyme-coding genes included in the sequential biosynthesis pathway were overexpressed in the fungus, penicillin production increased over 100-fold compared to the wild-type strain (Marui et al., 2010) (Figure 1B-①). It is also known that the genes implicated in the synthesis of some secondary metabolites compose gene clusters on chromosomes (Keller and Hohn, 1997; Brakhage and Schroeckh, 2011). In such clusters, one gene often encodes a transcription factor to regulate expression of the whole cluster (Brakhage, 2013). Therefore, overexpression of the transcription factor gene leads to overexpression of all the genes in the cluster, resulting in a massively increased production yield (Figure 1B-②). Well-established examples of such transcription factors include *kojR* in the kojic acid biosynthesis gene cluster of *A. oryzae* (Marui et al., 2011), the CtnR-like transcriptional activator gene in the asperfuranone biosynthesis gene cluster of *A. nidulans* (Chiang et al., 2009), and *apdR* in the aspyridone A/B biosynthesis gene cluster of *A. nidulans* (Bergmann et al., 2007). Furthermore, the *laeA* gene was found to encode a global regulator of secondary metabolite genes, and its overexpression increased the production of penicillin and lovastatin in *A. nidulans* (Bok and Keller, 2004). Overexpression of *laeA* in *A. oryzae* also triggered the expression of two clusters of heterologous biosynthetic genes (the monacolin K (MK) gene cluster of *Monascus pilosus* and the terrequinone A (TQ) gene cluster of *A. nidulans*), resulting in the production of the corresponding metabolite, MK or TQ (Sakai et al., 2012) (Figure 1B-③).

To predict which genes are involved in the synthesis of metabolites and enzymes as well as to estimate which genes are bottlenecks in their synthesis, biologists sometimes need to analyze the enormous dataset generated by both genome sequencing and subsequent various experiments in the “Omics,” i.e., transcriptome, metabolome, proteome, etc. However, many biologists do not seem to be well versed in bioinformatics and the associated tasks involving programming. Thus, it will be useful for them to master the usage of various software provided in packages or on websites, which interfaces them with bioinformatics. It will also be meaningful to collaborate with other scientists or specialists of genomic information analysis, statistical analysis, and enormous data processing.

The technology used to predict strategies appropriate for enhancing the production of primary metabolites has been established in the “Omics” research fields (Copeland et al., 2012; Tomar and De, 2013; Toya and Shimizu, 2013). For example, the COBRA toolbox is equipped with some programs like the flux balance analysis (FBA) (Varma and Palsson, 1994) involved in the technology. Any researcher can utilize the programs by accessing the COBRA toolbox via the MATLAB software package (Zomorrodi et al., 2012). Using these programs, the probability of creating mutant strains with increased ability for production would increase. Therefore, together with using the conventional “trial and error” strategy, it is considered best that researchers in the field also challenge the use of the microbial metabolism simulation technology, which will enable an increase in the yield of target primary metabolites in a more efficient manner.

## Conflict of interest statement

The author declares that the research was conducted in the absence of any commercial or financial relationships that could be construed as a potential conflict of interest.
